# Association between ABO Blood Group and Risk of Congenital Heart Disease: A 6-year large cohort study

**DOI:** 10.1038/srep42804

**Published:** 2017-02-17

**Authors:** Bailing Zu, Guoling You, Qihua Fu, Jing Wang

**Affiliations:** 1 Department of Laboratory Medicine, Shanghai East Hospital, Tongji University School of Medicine, Shanghai, China; 2 Department of Laboratory Medicine, Shanghai Children’s Medical Center, Shanghai Jiao Tong University School of Medicine, Shanghai, China

## Abstract

ABO blood group, except its direct clinical implications for transfusion and organ transplantation, is generally accepted as an effect factor for coronary heart disease, but the associations between ABO blood group and congenital heart disease (CHD) are not coherent by previous reports. In this study, we evaluated the the potential relationship between ABO blood group and CHD risk. In 39,042 consecutive inpatients (19,795 CHD VS 19,247 controls), we used multivariable logistic regression to evaluate the roles of ABO blood group, gender, and RH for CHD. The associations between ABO blood group and CHD subgroups, were further evaluated using stratification analysis, adjusted by gender. A blood group demonstrated decreased risk for isolated CHD (OR 0.82; 95% CI, 0.78–0.87) in individuals with A blood group in the overall cohort analysis, and the finding was consistently replicated in independent subgroup analysis. ABO blood group may have a role for CHD, and this novel finding provides ABO blood group as a possible marker for CHD, but more studies need to be done.

The human histo-blood group ABO system is crucial for clinical transfusion and transplantation medicine. ABO blood group consists of four types: A, B, AB, and O, determined by three alleles at ABO genetic locus^1^. A and B alleles encode A and B transferases, respectively, which transfer an N-acetylgalactosamine (GalNAc) or a galactose (Gal) to H substances. O allele encodes a truncated protein deprived of glycosyltransferase activity, which is triggered by a single nucleotide deletion (Δ261) in exon 6^2^. Glycosylation is one of the most common post-translational modifications mediated by enzymes, and it plays important roles in cellular communication during cell differentiation and development. Except the direct clinical implications for transfusion and organ transplantation compatibility, it is being increasingly recognized that ABO blood group allele is associated with risk of venous thromboembolic events, myocardial infarction, cerebrovascular ischemic events and coronary heart disease^3^, individuals with non-O blood groups being at a elevated risk compared to O blood group.

Congenital heart disease (CHD) defines a series of structural and functional defects of heart and great vessels arising during embryogenesis. CHD is the most common type of congenital disorder in newborns and accounts for one third of congenital anomalies^4^. International Classification of Disease codes^5^, ICD-10 lists the currently identified congenital heart defect subgroups, which designates specific anatomic or hemodynamic lesions.

CHD is often partitioned into isolated CHD and syndromic CHD. Isolated CHD occurs as an isolated defect (single or complex defect confined to the cardiovascular system), while syndromic CHD is characterized by heart defect combined with multiple malformations of other organ systems (e.g. neurodevelopmental, genitourinary, or immune system). Down syndrome is the most common developmental syndrome with prominent CHD phenotypes by chromosomal anomaly^6^, and about 40–50% of Down syndrome patients have a heart defect, such as complete atrioventricular canal defect (CAVC). Recent studies have elucidated that isolated CHD and syndromic CHD are triggered by different genetic and epigenetic mechanisms, respectively^6^. Thus, these two groups should not be compressed into one for analysis.

In 1969, *Brendemoen* first reported an excess of A blood group among Norway ventricular septal defect patients^7^, however, available reports failed to provide a coherent picture of the association between ABO blood group and CHD risk^8^,^9^. In order to clarify these issues, we utilized a large cohort consisting of 39,042 subjects to investigate the potential relationships between ABO blood group and CHD risk.

## Results

### Baseline characteristics of CHD patients and CHD-free controls

Basic characteristics of the population included in our study are presented in Table 1 (duplicated records of the same person identity being excluded for quality control). A total of 39,042 subjects (19,795 CHD VS 19,247 control) were included in the analysis, of whom 42.2% (n = 16,478) were females. The median age of the two groups at entry were both 1.8 years, and the Wilcoxon rank-sum test did not show any statistically significant difference between non-CHD and CHD groups (p = 0.054).

### ABO blood group and CHD risk

The analysis of 19,795 CHD included 13,067 individuals with non-O blood groups (5,375 for A, 5,731 for B, and 1,961 for AB) and 6,728 with O blood group. Based on precise diagnosis, 19,479 isolated CHD and 316 syndromic CHD were included. As shown in Table 2, no significant association for Rh positivity was found in patients with isolated CHD or syndromic CHD, when compared to the control population (p = 0.16). Moreover, neither ABO blood group nor gender was statistically significantly associated with syndromic CHD (p > 0.05). However, a significantly low odds ratio between A blood group with isolated CHD was observed, an OR of 0.82 (95% CI, 0.78–0.87) in individuals with A blood group compared to O blood group. Similarly, the analyses also suggested the gender influence the risk of isolated CHD, with lower OR in men (p < 0.001).

### ABO blood group and risk of isolated CHD subgroups

Further analyses of isolated CHD subgroups also revealed decreased risks for persons with A blood group. A consistent pattern was observed among all of the main isolated CHD subgroups (VSD, OR 0.78, p < 0.001; ASD, OR 0.82, p < 0.001; PDA, OR 0.81, p < 0.001; TOF, OR 0.84, p = 0.009; PA, OR 0.84, p = 0.036; DORV, OR 0.76, p = 0.003; TGA, OR 0.72, p = 0.001; COA, OR 0.71, p = 0.001), with relatively lower odds ratios for individuals with A blood group compared to those with O blood group (Table 3). However, no similar association for AB or B blood group was found in CHD subgroups (p > 0.05), except TOF subgroup. The risk patterns were largely similar across the subgroups, except somewhat higher ORs of AB blood group (OR 1.45; 95% CI, 1.23–1.71) and B blood group (OR 1.18; 95% CI, 1.04–1.33) for TOF subgroup.

Gender demonstrated a significant interaction for isolated CHD in both the overall and stratified analysis. For VSD, ASD and PDA subgroups, males exhibited lower risks (VSD, OR 0.94, p = 0.025; ASD, OR 0.70, p < 0.001; PDA, OR 0.62, p < 0.001). In contrast, males exhibited a higher risk for TGA subgroup (OR 1.61; 95% CI, 1.37–1.90).

## Discussion

Using a large cohort comprising 39,042 subjects (19,795 CHD VS 19,247 control), we were able to show that individuals with A blood group are at decreased risk of isolated CHD, compared to individuals with non-A blood group, and gender also demonstrated a significant interaction for isolated CHD.

Congenital heart disease, defined as structural and functional defects that arise during cardiac embryogenesis, triggered by genetic anomalies, environmental teratogens, maternal exposures and infectious agents^10^,^11^. Several studies explored the association between ABO blood type and CHD^7^,^8^,^9^. Since the exact pathophysiological mechanisms of most congenital cardiac disease remains to be unknown nowadays, it would be of value to demonstrate whether relationships between CHD and ABO blood phenotypes exist and whether the relationshipas further could be a potential marker for clinical prevention. However, the results of these previous reports were inconsistent, the conflicted findings of which may be largely due to the small sample sizes of these studies, but may also reflect genetic drift in natural selection for different populations. Our study has several important strengths. To our knowledge, It is by far the first study ever to have investigated the association between ABO blood group and CHD risk in such a large, well-defined cohort. Furthermore, the complete data from clinical data repository of the hospital, for transfusion management, ensured the reliability of the study.

Although the mechanism underlying the relationship between blood type and CHD is unknown, there are several possible explanations. The ABO gene locus is located on chromosome 9q34, and the gene products of the ABO system are glycotransferases with GalNAc (A)/Gal (B) specificity, which catalyze the transfer of carbohydrates to the H antigen, a precursor of ABO antigens. NOTCH1 and EHMT1, which are located on chromosome 9q34 near the ABO locus, play crucial roles in cardiovascular development^12^,^13^,^14^. NOTCH1 functions in the development of the semilunar valves and cardiac outflow tract, and deletion of EHMT1 manifests distinct features of 9q subtelomeric deletion syndrome or Kleefstra syndrome. Genetic inheritance may help explain the relationship between ABO blood type and CHD^15^. If multi-allelic SNPs, indels, and a diverse set of structural variants occur to NOTCH1 and EHMT1 loci of the haplotype at the stage of meiosis, significant associations could be detected between ABO allele frequencies and CHD for the local linkage disequilibrium of 9q34. Additionally, Hh signaling activation is positively associated with cardiac development^16^, and GALNT1 (N-acetylgalactosaminyltransferases1) mediated O-linked glycosylation of Shh serves as an essential process for its activation^17^. Thus, CHD risk could be decreased, while the biological activities of GALNT1 is altered by glycotransferases of A allele by competitive inhibition.

Our findings are less likely to be false positive because of consistent replications in overall cohort analysis and independent subgroup analysis. Also, inclusion and exclusion criteria for the case-control study were performed to minimize bias. In interpreting the results, some limitations of this study should be addressed. First, the study was performed just in chinese population. Though the included patients being from across the country, selection pressures cause diversified distribution of ABO genotype across different ethnic groups and different countries, thus larger studies covering multi-racial participants might provide more definitive conclusions to clarify the possible role of ABO. Second, 381 ABO alleles are reported in the Blood Group Antigen Gene Mutation Database (BGMUT, http://www.ncbi.nlm.nih.gov/projects/gv/rbc), which constitutes a great many ABO subtypes (e.g. A1, A2, A3, Aw, Ax, Ael). The included participants of the study were identified and classified by A, B, AB, and O blood group, and the data were analyzed in the heterogeneous mixture of ABO subtype. Further studies should include ABO subtype to investigate the association between ABO subtype and CHD, which may provide more precise value and reliable data.

Here by with this study, we confirmed that ABO blood group might be associated with isolated CHD risk, what’s more, gender demonstrates a significant interaction for CHD. Given that A blood group confer an overall decreased risk to all the main isolated CHD subgroups (VSD, ASD, PDA, TOF, PA, DORV, TGA, COA), ABO blood group may have a role in CHD risk and could potentially be added to available clinical prediction systems. This novel finding provides a potential mechanistic insight into the pathogenesis of CHD. However, studies with larger sample sizes covering multi-races should be further conducted to investigate its clinical utility in the future.

## Methods

### Study population

Data for this study were collected from Shanghai Children’s Medical Center, Shanghai Jiao Tong University School of Medicine. The included subjects of the study were all the consecutive and eligible inpatients with identified ABO blood group and newly diagnosed with established disease (CHD or other diseases) in Shanghai Children’s Medical Center, registered between March 2010 and May 2016. Major exclusion criteria for the study were as follows: inpatients with no established diagnosis, detailed medical history or ABO type; inpatients with tumor, leukemia or organ transplantation record (these factors can alter ABO phenotype at different stages of disease^18^,^19^,^20^,^21^); duplicated electronic records.

Informed consent was obtained from all subjects. All experimental protocols were approved by the Ethics Committee of the Shanghai Children’s Medical Center and carried out in accordance with relevant guidelines.

### Study design

The primary objective was to analyze the association between ABO blood group and risk of CHD and CHD subgroups. Congenital heart defects generally are diagnosed by physical exam, echocardiography, electrocardiogram, chest x-ray, pulse oximetry and cardiac catheterization^22^. We used International Classification of Diseases, 10th version (ICD-10) diagnosis codes (Q20-Q28) to classify CHD anatomic subgroups. ABO blood group of the individual was confirmed by standard serological techniques and genotyping.

### Statistical analysis

A logistic stepwise regression analysis was used to assess the frequencies in gender and RH between the cases and the CHD-free controls. A Wilcoxon rank-sum (Mann-Whitney) test was used to compare the age of two groups. The associations between ABO blood group and CHD subtypes, were evaluated using unconditional logistic regression analysis. All statistical calculations were performed with STATA 12.0 for Windows (Chicago, IL). A p value of <0.05 was considered statistically significant.

## Additional Information

**How to cite this article**: Zu, B. *et al*. Association between ABO Blood Group and Risk of Congenital Heart Disease: A 6-year large cohort study. *Sci. Rep.* **7**, 42804; doi: 10.1038/srep42804 (2017).

**Publisher's note:** Springer Nature remains neutral with regard to jurisdictional claims in published maps and institutional affiliations.

## Figures and Tables

**Table 1:** Characteristics of study population.

	Non-CHD		CHD	
No. subjects, N (% of total)	19247	(49.3)	19795	(50.7)
Female, N (%)	7728	(40.2)	8750	(44.2)
Age at study entry, N (%)				
<1 years	8619		9260	
1–3 years	3411		5228	
≥3 years	7217		5307	
Mean age, years (IQR)	1.8	(0.06–5)	1.8	(0.04–3)
ABO blood group, N (%)				
A	5962	(31.0)	5375	(27.2)
AB	1742	(9.1)	1961	(9.9)
B	5400	(28.1)	5731	(29.0)
O	6143	(31.9)	6728	(34.0)
Rhesus antigen positivity, N (%)	19158	(99.5)	19723	(99.6)
classification of CHD, N (%)				
isolated CHD	NA		19479	(98.4)
syndromic CHD	NA		316	(1.6)

CHD, congenital heart disease; IQR, interquartile range; NA, not avaiable.

**Table 2:** Risk of congenital heart disease in relation to blood group.

Classification	ABO blood group								gender (M vs F)	RH (P vs N)
	A		B		AB		O			
	case	OR (95% CI)	case	OR (95% CI)	case	OR (95% CI)	case	OR (95% CI)	case	OR (95% CI)
isolated CHD	5284	0.82 (0.78–0.87)	5647	0.97 (0.92–1.02)	1938	1.04 (0.96–1.11)	6610	1.00 (ref)	0.81 (0.84–0.88)	1.25 (0.92–1.71)
syndromic CHD	91	0.80 (0.60–1.05)	84	0.81 (0.61–1.07)	23	0.69 (0.44–1.08)	118	1.00 (ref)	0.97 (0.78–1.22)	1.45 (0.20–10.43)

CHD, congenital heart disease.

**Table 3:** Risk of isolated congenital heart disease subgroups in relation to blood group, presented overall and stratified by sex.

	ABO blood group							
	A		B		AB		O	
	case	OR (95% CI)	case	OR (95% CI)	case	OR (95% CI)	case	OR (95% CI)
**VSD**								
Overall	2425	0.78 (0.73–0.83)	2818	1.00 (0.94–1.07)	964	1.06 (0.97–1.16)	3192	1.00 (ref)
Male	1414	0.79 (0.73–0.86)	1667	1.03 (0.96–1.11)	574	1.10 (0.99–1.23)	1840	1.00 (ref)
Female	1011	0.77 (0.70–0.84)	1151	0.97 (0.89–1.06)	390	1.02 (0.90–1.15)	1352	1.00 (ref)
**ASD**								
Overall	1698	0.82 (0.76–0.88)	1825	0.97 (0.90–1.05)	643	1.06 (0.96–1.18)	2134	1.00 (ref)
Male	858	0.82 (0.74–0.90)	929	0.98 (0.89–1.08)	341	1.11 (0.98–1.27)	1079	1.00 (ref)
Female	840	0.82 (0.74–0.90)	896	0.97 (0.88–1.06)	302	1.00 (0.88–1.16)	1055	1.00 (ref)
**PDA**								
Overall	838	0.81 (0.73–0.89)	905	0.97 (0.88–1.06)	302	1.00 (0.87–1.15)	1066	1.00 (ref)
Male	376	0.77 (0.67–0.89)	472	1.07 (0.94–1.22)	143	1.00 (0.83–1.22)	501	1.00 (ref)
Female	462	0.84 (0.74–0.96)	433	0.87 (0.77–0.99)	159	0.99 (0.83–1.19)	565	1.00 (ref)
**TOF**								
Overall	431	0.84 (0.73–0.96)	548	1.18 (1.04–1.33)	218	1.45 (1.23–1.71)	530	1.00 (ref)
Male	254	0.77 (0.65–0.90)	332	1.10 (0.95–1.29)	139	1.43 (1.17–1.76)	342	1.00 (ref)
Female	177	0.97 (0.79–1.19)	216	1.31 (1.07–1.59)	79	1.48 (1.13–1.94)	188	1.00 (ref)
**PA**								
Overall	271	0.84 (0.71–0.99)	285	0.97 (0.83–1.15)	108	1.14 (0.91–1.43)	333	1.00 (ref)
Male	152	0.77 (0.62–0.95)	193	1.08 (0.89–1.32)	56	0.97 (0.72–1.31)	203	1.00 (ref)
Female	119	0.94 (0.73–1.21)	92	0.81 (0.61–1.05)	52	1.41 (1.02–1.95)	130	1.00 (ref)
**DORV**								
Overall	215	0.76 (0.64–0.91)	224	0.88 (0.73–1.05)	83	1.00 (0.78–1.29)	291	1.00 (ref)
Male	136	0.77 (0.62–0.97)	139	0.87 (0.70–1.09)	54	1.05 (0.77–1.43)	181	1.00 (ref)
Female	79	0.74 (0.55–0.99)	85	0.88 (0.66–1.17)	29	0.93 (0.62–1.40)	110	1.00 (ref)
**TGA**								
Overall	175	0.72 (0.59–0.88)	205	0.93 (0.77–1.13)	82	1.16 (0.90–1.49)	250	1.00 (ref)
Male	116	0.68 (0.53–0.86)	150	0.97 (0.77–1.20)	60	1.20 (0.89–1.60)	177	1.00 (ref)
Female	59	0.83 (0.59–1.18)	55	0.86 (0.60–1.22)	22	1.06 (0.66–1.72)	73	1.00 (ref)
**COA**								
Overall	172	0.71 (0.58–0.87)	196	0.90 (0.74–1.08)	72	1.02 (0.78–1.33)	249	1.00 (ref)
Male	112	0.72 (0.57–0.92)	117	0.83 (0.65–1.06)	47	1.04 (0.75–1.44)	160	1.00 (ref)
Female	60	0.69 (0.50–0.97)	79	1.00 (0.74–1.37)	25	0.99 (0.63–1.55)	89	1.00 (ref)

VSD, ventriculap septal defect; ASD, atrial septal defect; PDA, patent ductus arteriosus; TOF, tetralogy of Fallot; PA, pulmonary atresia; DORV, double outlet right ventricle; TGA, transposition of the great arteries; COA, coarctation of aorta.

## References

[b1] Yamamoto F. . An integrative evolution theory of histo-blood group ABO and related genes. Sci Rep 4, 6601 (2014). 25307962 10.1038/srep06601 PMC5377540

[b2] Itou M. . Analysis of a Larger SNP Dataset from the HapMap Project Confirmed That the Modern Human A Allele of the ABO Blood Group Genes Is a Descendant of a Recombinant between B and O Alleles. Int J Evol Biol 2013, 406209 (2013). 24288652 10.1155/2013/406209 PMC3830805

[b3] Vasan S. K. . ABO Blood Group and Risk of Thromboembolic and Arterial Disease: A Study of 1.5 Million Blood Donors. Circulation 133, 1449–1457, discussion 1457 (2016). 26939588 10.1161/CIRCULATIONAHA.115.017563

[b4] Mozaffarian D. . Heart Disease and Stroke Statistics-2016 Update: A Report From the American Heart Association. Circulation 133, e38–360 (2016). 26673558 10.1161/CIR.0000000000000350

[b5] Tanno L. K. . Smoothing the transition from International Classification of Diseases, Tenth Revision, Clinical Modification to International Classification of Diseases, Eleventh Revision. J Allergy Clin Immunol Pract (2016). 10.1016/j.jaip.2016.06.024 27546357

[b6] Blue G. M. . Congenital heart disease: current knowledge about causes and inheritance. Med J Aust 197, 155–159 (2012). 22860792 10.5694/mja12.10811

[b7] Brendemoen O. J. ABO blood-groups in patients with septal defects. Lancet 2, 1140 (1969). 10.1016/s0140-6736(69)90739-9 4188082

[b8] Odegard K. C. . Distribution of ABO phenotypes in patients with congenital cardiac defects. Cardiol Young 18, 307–310 (2008). 18460227 10.1017/S104795110800231X

[b9] Nasri A. . [Distribution of ABO groups in patients undergoing heart surgery]. Orv Hetil 134, 1027–1031 (1993). 8493031

[b10] Fahed A. C. . Genetics of congenital heart disease: the glass half empty. Circ Res 112, 707–720 (2013). 23410880 10.1161/CIRCRESAHA.112.300853 PMC3827691

[b11] Wilson K. D. . A Rapid, High-Quality, Cost-Effective, Comprehensive and Expandable Targeted Next-Generation Sequencing Assay for Inherited Heart Diseases. Circ Res 117, 603–611 (2015). 26265630 10.1161/CIRCRESAHA.115.306723 PMC4568077

[b12] Koenig S. N. . Endothelial Notch1 Is Required for Proper Development of the Semilunar Valves and Cardiac Outflow Tract. J Am Heart Assoc 5 (2016). 10.1161/JAHA.115.003075 PMC4843530 27107132

[b13] Priest J. R. . De Novo and Rare Variants at Multiple Loci Support the Oligogenic Origins of Atrioventricular Septal Heart Defects. PLoS Genet 12, e1005963 (2016). 27058611 10.1371/journal.pgen.1005963 PMC4825975

[b14] Vargiami E. . Multiple Coronary Artery Microfistulas in a Girl with Kleefstra Syndrome. Case Rep Genet 2016, 3056053 (2016). 27239352 10.1155/2016/3056053 PMC4867054

[b15] Wall J. D. & Stevison L. S. Detecting Recombination Hotspots from Patterns of Linkage Disequilibrium. G3 (Bethesda) 6, 2265–2271 (2016). 27226166 10.1534/g3.116.029587 PMC4978882

[b16] Zhang K. K. . Gene network and familial analyses uncover a gene network involving Tbx5/Osr1/Pcsk6 interaction in the second heart field for atrial septation. Hum Mol Genet 25, 1140–1151 (2016). 26744331 10.1093/hmg/ddv636 PMC4764195

[b17] Li C. . GALNT1-Mediated Glycosylation and Activation of Sonic Hedgehog Signaling Maintains the Self-Renewal and Tumor-Initiating Capacity of Bladder Cancer Stem Cells. Cancer Res 76, 1273–1283 (2016). 26676748 10.1158/0008-5472.CAN-15-2309

[b18] Koestner S. C. . Histo-blood group type change of the graft from B to O after ABO mismatched heart transplantation. Lancet 363, 1523–1525 (2004). 15135601 10.1016/S0140-6736(04)16179-5

[b19] Bianco-Miotto T. . DNA methylation of the ABO promoter underlies loss of ABO allelic expression in a significant proportion of leukemic patients. PLoS One 4, e4788 (2009). 19274076 10.1371/journal.pone.0004788 PMC2650780

[b20] Chihara Y. . Loss of blood group A antigen expression in bladder cancer caused by allelic loss and/or methylation of the ABO gene. Lab Invest 85, 895–907 (2005). 15880137 10.1038/labinvest.3700268

[b21] Cohen D. . Red blood cell alloimmunization in transfused patients with bone marrow failure syndromes. Transfusion 56, 1314–1319 (2016). 27080340 10.1111/trf.13608

[b22] Perloff J. K. & Marelli A. J. Clinical recognition of congenital heart disease[M]. Elsevier Health Sciences (2012).

